# Existing food processing classifications overlook the phytochemical composition of processed plant-based protein-rich foods

**DOI:** 10.1038/s43016-025-01148-5

**Published:** 2025-03-24

**Authors:** Jasmin Raita, Hany Ahmed, Kang Chen, Veera Houttu, Retu Haikonen, Anna Kårlund, Maaria Kortesniemi, Baoru Yang, Ville Koistinen, Kati Hanhineva

**Affiliations:** 1 Food Sciences Unit, Department of Life Technologies, University of Turku, Turku, Finland; 2 College of Food Science and Engineering, Ningbo University, Ningbo, PR China; 3 Nutrition and Food Research Center, Faculty of Medicine, University of Turku, Turku, Finland; 4 Institute of Public Health and Clinical Nutrition, School of Medicine, University of Eastern Finland, Kuopio, Finland

**Keywords:** Metabolomics, Metabolomics

## Abstract

According to existing food processing classification systems, plant-based protein-rich (PBPR) foods are often considered ‘ultra-processed’—and therefore perceived as unhealthy—despite their ability to provide various bioactive compounds beneficial for human health. Here we used a non-targeted metabolomics approach to analyse the impact of processing on the biochemical composition of PBPR foods. Our results show that existing food classification systems may provide questionable categories for PBPR foods without considering their overall biochemical composition, including phytochemicals. An analysis focusing specifically on biochemical compounds of soy-based products manufactured using various technologies showed no clear distinctions between processing groups in the principal component analysis based on the NOVA and Poti classification. However, clear differences were found between soy-based products based on their phytochemical profile. Although food processing classification systems are welcome in their attempt to guide consumers towards healthy choices, they should be improved to more accurately reflect the biochemical composition of PBPR foods.

## Abstract (web summary)

Although plant-based protein-rich (PBPR) foods contain nutritionally bioactive compounds, they are often classified as ultra-processed foods, which most consumers perceive as unhealthy. Using a non-targeted metabolomic approach, this study shows that existing food classification systems do not consider the biochemical composition of PBPR foods, potentially misleading consumers to avoid these products.

## Main

The majority of food we consume undergoes some degree of processing. Industrial food processing entails a wide range of techniques, from preserving whole plant materials to producing foods prepared from isolated components such as sugars, oils and proteins. In this context, concern has arisen regarding food items produced by extensive technological processes, commonly referred to as ‘ultra-processed’ foods, due to their association with various health issues, including poor cardiometabolic health and obesity^1–3^. Currently, intensive debate is ongoing among various stakeholders involving academia, the food industry, regulatory authorities and consumers regarding the appropriate classification of food processing techniques in relation to their health implications. As concluded in a recent study^4^, science-based classifications and clinical research are essential to address the discrepancies regarding food product classifications and the impact of differentially processed food on human health.

The NOVA classification system^5^ has existed since 2009^6^ and is widely used in the literature to describe food processing, categorizing foods into four groups: ‘unprocessed and minimally processed’, ‘processed culinary ingredients’, ‘processed’ and ‘ultra-processed’. NOVA defines ultra-processed foods as industrial formulations containing, for example, additives for flavour or colour enhancement^5^. Other classification methods, such as that in Poti et al.^7^, use categories based on the alterations made to the product, including ‘unprocessed/minimally processed’, ‘basic processed’, ‘moderately processed’ and ‘highly processed’. The current classification systems mainly focus on the technological processes and the addition of ingredients, but they do not consider what is lost due to processing, that is, the level of refinement.

The classification of processed foods using the existing systems is problematic from a health perspective, as certain (ultra-)processed foods have been associated with adverse health effects^8^, but not all of them. For example, a study^9^ showed that plant-based foods, even when classified as ultra-processed by NOVA, were not associated with increased risk for cancer and cardiometabolic diseases; only the meat-based products and artificial and sugar-sweetened beverages were linked to such risks. Various processing techniques, even those that cause major structural and biochemical changes in the food, are not necessarily linked with adverse health effects. Whole-grain bread would fall within the processed or even ultra-processed category according to the NOVA classification due to the inclusion of multiple species of whole grain or added ingredients, such as salt, which disregards its various health benefits^10,11^ and its high content of fibre and bioactive phytochemicals^12^. The mechanisms by which the currently applied food processing methods alter the nutritional and biochemical properties and thereby influence the risk of chronic diseases are not comprehensively addressed. Ultimately, it all comes down to the question: what does the food contain when it is ingested? The processing technique itself does not have an impact on human health; rather, it is the resulting food matrix and its embedded biochemical composition that affect outcomes. Indeed, the nutritional content and biochemical composition are important factors when considering the healthiness of foods, but the existing classification systems often overlook these aspects. Despite this, these systems are still used to influence the food choices of consumers.

Currently, people are encouraged to increase the consumption of plant-based foods as a replacement for meat owing to their environmental sustainability and health benefits^13,14^. Diets favouring plant-based foods, including vegetables, fruits, whole grains, legumes, vegetable oils, nuts and seeds, have shown health-promoting effects such as reduced risk for cardiovascular diseases^15–17^ due to their dietary fibre, vitamins, minerals, phytochemicals, unsaturated fatty acids and low level of saturated fat. However, concern has emerged regarding new plant-based protein-rich (PBPR) foods designed to resemble meat organoleptically, the so called meat analogues. Their nutritional quality and health effects can vary, while consumers may presume that the plant-based options are automatically healthier^18^. Therefore, there is a growing demand to study the biochemical composition linked to health effects resulting from various industrial processes to provide reliable classifications to consumers.

Here our objective was to exemplify the impact of various food processing technologies on the biochemical composition of PBPR food products by focusing on phytochemicals. We applied non-targeted metabolomics using liquid chromatography coupled with mass spectrometry (LC–MS) on a large variety of PBPR food products, focusing particularly on soy-based products and their isoflavonoid composition as an example, and evaluated the findings with regard to the existing processing classification systems. We hypothesize that the biochemical profile affected by processing is an important determinant that may be linked to diet-related health outcomes and should therefore be considered when formulating classification systems for processed foods.

## Results

### Biochemical composition of various protein-rich food products

**Fig. 1:**
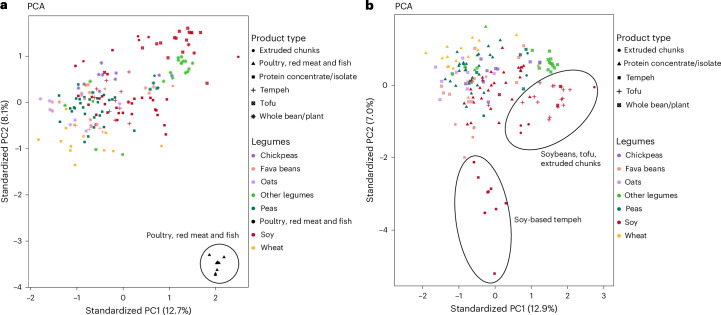
Differences in the biochemical compositions of various food products. **a**, Differences in PBPR food products and unseasoned poultry, red meat and fish products (*n* = 176) are shown using PCA of molecular features (*n* = 9,389) obtained from non-targeted LC–MS-based metabolite profiling analysis. **b**, Differences in PBPR food products (*n* = 168) are shown using PCA of molecular features (*n* = 9,389) obtained from non-targeted LC–MS-based metabolite profiling analysis. PC1, principal component 1; PC2, principal component 2. The variance explained by each principal component is indicated by the percentages on the *x* and *y* axes.

We performed a non-targeted LC–MS metabolomics examination on the biochemical composition of 168 PBPR food products, ranging from whole legumes to products made from protein concentrates or isolates, alongside 8 unseasoned and unprocessed poultry, red meat and fish products (2 minced beef products, 2 chicken strip products, 2 salmon fillets and 2 pork strips) as controls. The nutritional information for plant- and animal-based products is provided in Supplementary Table 1. Principal component analysis (PCA) of the complete metabolomics data (Fig. 1a) distinctly separated PBPR foods made from chickpeas, fava beans, oats, peas, soy, wheat, other legumes (including whole legumes such as black beans, white beans, kidney beans, red lentils, butter beans, green lentils and borlotti beans, and three products made with rice and sunflower seeds) from the unseasoned poultry, red meat and fish. When further examining the PBPR foods solely by PCA (Fig. 1b) and *t*-distributed stochastic neighbour embedding analysis (Supplementary Fig. 1), their clustering reflected not only the raw material used in their production but also their product types (Supplementary Table 2), such as tofu, tempeh, extruded chunks, whole legumes and products made from protein concentrates or isolates. Notably, soy-based products including tempeh, tofu, extruded chunks and whole legumes were clustered separately from other PBPR foods.

### Impact of various processing techniques on soy-based products

**Fig. 2:**
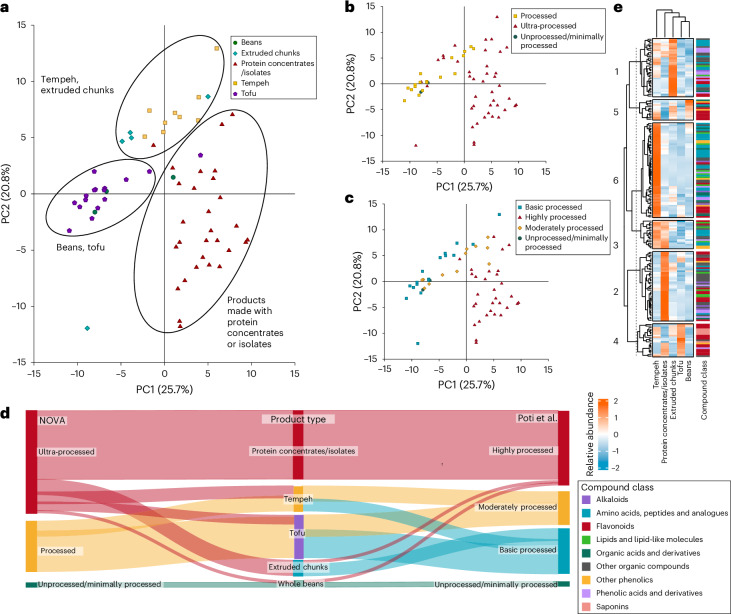
The impact of processing on the biochemical composition of variously processed soy-based products. **a**, Soy-based products classified according to product type, with PCA performed on identified compounds (*n* = 193). **b**, Soy-based products classified according to the NOVA classification system, with PCA performed on identified compounds (*n* = 193). **c**, Soy-based products classified according to the Poti et al.^7^ classification system, with PCA performed on identified compounds (*n* = 193). The variance explained by each principal component is indicated by the percentages on the *x* and *y* axes. **d**, Soy-based products classified according to product type, the NOVA classification system and the Poti et al.^7^ classification system, illustrated with a Sankey diagram. **e**, Identified compounds (*n* = 193) in soy-based products visualized using *k*-means clustering (*z*-normalized; numbers represent clusters 1–6).

Next, we focused on the soy-based products (*n* = 62), due to soy’s popularity as a raw material in plant-based products and its representation across various food categories processed with varying techniques. A total of 193 compounds, belonging to classes such as flavonoids and phenolic acids, were identified in these products (Supplementary Data 1). The PCA (Fig. 2a) shows the effect of processing on the biochemical composition of soy-based products, forming three distinct clusters that separate beans and tofu, tempeh and extruded chunks, and products made with protein concentrates or isolates. When existing classification systems were used for these products (Fig. 2b,c), clear distinctions between the groups were not observed. Ultra-processed products were located next to unprocessed or minimally processed products using the NOVA classification system (Fig. 2b). Similarly, using the Poti et al.^7^ classification system (Fig. 2c), moderately processed products were found next to the unprocessed or minimally processed ones.

The Sankey diagram (Fig. 2d) illustrates the categorization of soy-based products analysed in this study according to the NOVA and Poti et al.^7^ systems. In the middle of the diagram, products are categorized as product type based on the protein type used, that is, tempeh, extruded chunks, tofu, whole beans and products made with protein concentrates or isolates. As seen from the figure, certain product types fall into two different categories. In NOVA, whole beans are considered as unprocessed or minimally processed, except for one whole-bean product, which is a burger steak. Tofu and tempeh are considered as processed products, except for those in the ultra-processed category, which are pre-fried and seasoned. Products made with protein concentrates or isolates with several added ingredients and extruded chunks are considered ultra-processed. According to the Poti et al.^7^ classification system, the same whole bean categorized as ultra-processed in NOVA and one product made with extruded chunks fell into the highly processed category. Tofu and tempeh vary from basic processed to moderately processed, whereas products made with protein concentrates or isolates are highly processed.

The results from a clustering analysis with the identified compounds (*n* = 193) are presented in Fig. 2e. Compounds distinguishing extruded chunks from other product types in cluster 1 (Fig. 2e) include acetyl derivatives of isoflavonoids. Cluster 2 includes compounds obtained from spices, such as the black-pepper-derived alkaloids piperine, piperanine and piperolein B^19^. Cluster 3 includes curcumenol derived from turmeric^20^, shogaol from ginger^21^ and capsaicin from chilli peppers^21^. Products made with protein concentrates or isolates and tofu contain compounds derived from spices^22^, such as rosmarinic acid and cirsimaritin, and are combined in cluster 4. In addition, saponins and some forms of isoflavonoids were present in tofu, and in lesser amounts in tempeh and extruded chunks in the same cluster. In cluster 5, beans, extruded chunks and tempeh include malonyl derivatives of isoflavonoids and other flavonoids. Cluster 6 contained isoflavonoid aglycones, amino acids and peptides, and other compounds derived from the fermentation process, such as 3-hydroxyanthranilic acid and 3-hydroxymethylglutaric acid^23^.

### Isoflavonoids in soy-based products

**Fig. 3:**
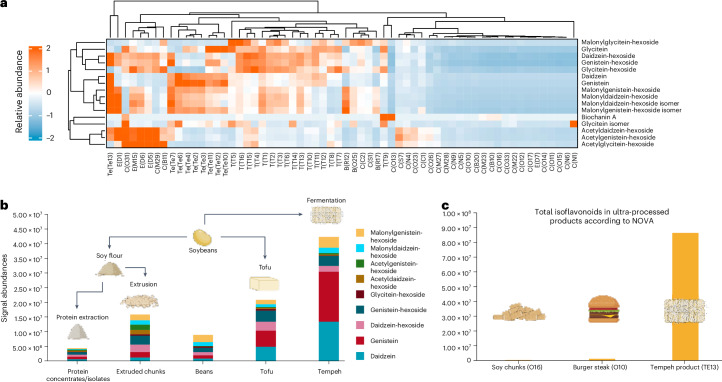
Relative abundances of isoflavonoids in various products processed from soybeans. **a**, Relative abundances (*z*-normalized) of isoflavonoids in individual soy-based products expressed in a heatmap. Sample codes are shown in parentheses (Te, tempeh; E, extruded chunks; T, tofu; C, protein concentrates/isolates; B, beans). **b**, The differences between isoflavonoid abundances in different product types are shown. **c**, Total isoflavonoid abundances in three different soy-based products categorized as ultra-processed according to NOVA. Panels **b** and **c** created with BioRender.com.

When focusing more closely on phytochemical composition, isoflavonoids were the key class of compounds in the soy products affected by various processing techniques (Fig. 3 and Supplementary Fig. 2). The abundance of isoflavonoids was low in nuggets, minced and pulled products, and steak and other products, which are made using protein concentrates or isolates (Fig. 3a). Acetyl derivatives of daidzein-hexoside, genistein-hexoside and glycitein-hexoside were mainly present in extruded chunks and other products made with extrusion. Malonyl and hexoside derivatives were found in whole beans, tofu and extruded chunks, whereas the aglycone forms daidzein, genistein and glycitein were most abundant in tempeh (Fig. 3b and Supplementary Fig. 3). Here we have focused on the identified compounds that seem to be more abundant in foods after processing compared with whole soybeans. However, some unidentified derivatives of isoflavonoids might still be present in whole soybeans, and some compounds may be strongly bound to the fibre matrix, making them more difficult to extract. One tempeh product was abundant in isoflavonoids, despite being categorized as ultra-processed in the NOVA system, similar to the soy chunk and burger steak products made from purified proteins that were nearly devoid of phytochemicals (Fig. 3c).

## Discussion

**Fig. 4:**
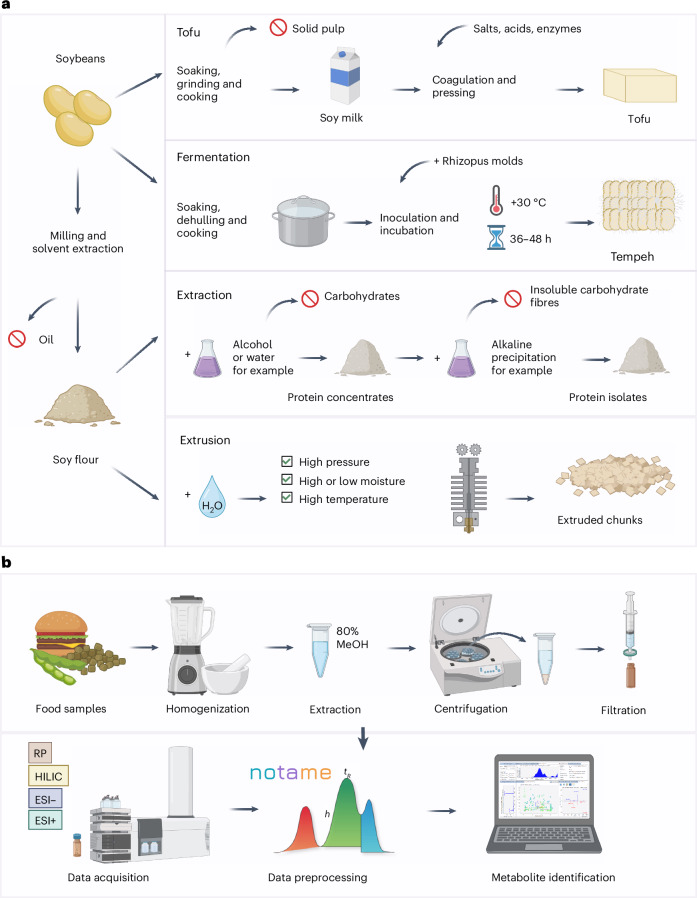
Diagrams illustrating soybean processing and non-targeted metabolomics analysis using LC–MS. **a**, Common processing techniques used for soybeans, including the preparation of tofu, tempeh, extruded chunks, and protein concentrates or isolates. **b**, The non-targeted LC–MS metabolomics approach used in this study, illustrating the sample preparation, data acquisition and analysis. LC using RP and HILIC coupled with QTOF-MS with positive and negative ionizations. Figure created with BioRender.com. ESI+, electrospray ionization positive mode; ESI−, electrospray ionization negative mode.

In this study, we showed the effect of various processing techniques (Fig. 4) on the biochemical composition of PBPR foods and the limitations of existing food processing classification systems in categorizing them. Our approach, using non-targeted metabolomics, focused on the biochemical composition of PBPR products, particularly the phytochemicals, which are traditionally not considered as nutrients despite providing various health benefits^24^. We highlighted the effect of processing by using soy isoflavonoids as an example, and showed that fermentation increased their abundances, whereas foods made with refined protein concentrates or isolates clearly had diminished abundances of these phytochemicals. By leaving out consideration of the biochemical content of foods, existing food processing classification systems fail in several cases to provide a meaningful interpretation of the effect of processing on the food product. These systems often categorize products containing beneficial bioactive compounds as processed or ultra-processed, potentially misleading consumers into avoiding them^25^.

All PBPR foods analysed in this study, including products made with protein concentrates or isolates, differed from the unseasoned and unprocessed poultry, red meat and fish in their biochemical composition, as shown in the PCA (Fig. 1a). In addition, the main raw material of PBPR foods led to clear separation in the PCA due to the different genetic backgrounds of the plant species that determine their phytochemical profiles (Fig. 1b). Furthermore, the processing technique used impacted the composition, which was particularly evident for the various soy-based foods. As soy is one of the most used raw materials in plant-based foods^26^, we decided to focus on its biochemical composition in more detail.

The effect of processing on soy-based products is evident in the PCA (Fig. 2a), where whole beans and tofu were separated from extruded chunks, tempeh, and products made with protein concentrates or isolates. This clustering reflects differences in the biochemical compositions of these soy-based products due to processing, such as the loss of beneficial bioactive compounds, as observed in previous studies^27,28^. The one outlier sample (extruded chunk) in the PCA (Fig. 2a) suggests that the extrusion technique used may somehow differ from the techniques used in other extruded chunks. Therefore, it is crucial to understand the impact of processing on these products, as they may appear similar based on the nutritional label despite having clear differences in their biochemical composition and, consequently, their nutritional value. When the NOVA and Poti classification systems were used to categorize these products in the PCA (Fig. 2b,c), different classifications clustered next to each other, and a clear separation was not observed. This may result from the addition of ingredients such as herbs and spices, which can beneficially influence the biochemical composition and nutritional value, whereas the classification is often shifted to a more processed level. For instance, some meat analogues like burger steaks contain added colourants, such as beetroot extract, to resemble the redness in meat^29^. However, meat analogues often contain high amounts of added unhealthy ingredients, such as saturated fat and sodium, to make them more palatable and acceptable for consumers^5^. Therefore, it would be useful for the food industry to have clear guidelines on added sodium and other unhealthy ingredients in plant-based products.

Bioactive compounds, such as isoflavonoids, might be lost during the several steps of protein extraction (Fig. 3a). Previously, it has been observed that roasting and extrusion can decrease the quantity of isoflavonoid malonyl derivatives while increasing acetyl derivatives, leading to a reduction in the total amount of isoflavonoids^28^. Similarly, the amount of phenolic compounds can decrease during the heating and preparation of tofu^27^. In soybeans, isoflavonoids are mainly found as malonyl derivatives (Fig. 3b), which are considered the stable storage forms^30^. The low isoflavonoid content in these whole soybeans most likely results from them being edamame beans, which are harvested for consumption before fully ripening, or from the inefficiency of extracting these compounds from the fibre matrix. The maturity of soybeans can have an impact on the total amount of isoflavonoids and other compounds, such as carotenoids^31^. Products made from soy flour, including extruded chunks and some other products, had higher abundances of isoflavonoids compared with whole soybeans. In extruded chunks, these isoflavonoids are mostly present as acetyldaidzein-hexoside, acetylgenistein-hexoside and acetylglycitein-hexoside, resulting from the extrusion. In addition, tofu and tempeh products contained various forms of isoflavonoids, whereas products made with protein concentrates or isolates seemed to contain less. Notably, tempeh had the highest abundance of aglycone forms of isoflavonoids (Fig. 3b), which is attributed to the β-glucosidase activity during fermentation causing the sugar unit to cleave off^32^. Aglycone forms, such as genistein, daidzein and glycitein, have increased bioavailability as they can be more readily absorbed because they do not need to be hydrolysed in the small or large intestines^33^. Therefore, the health benefits of tempeh consumption are commonly associated with isoflavonoids, as they are present in greater quantities and in a more bioavailable form compared with other soy products^34,35^. Tempeh was also the product with the highest total abundance of identified isoflavonoids, which may be due to the fact that, among the differentially processed soy-based products, tempeh is the ‘least refined’, that is, the whole bean matrix is used in the production process without removing any components. In addition to increasing the abundances of isoflavonoid aglycones in tempeh, fermentation can increase the amount of free amino acids and peptides^36,37^.

Whereas frying and other cooking methods may alter the chemical composition^38^, the addition of functional ingredients, such as herbs, does not necessarily compromise nutritional value, yet this can still result in the product being classified as ultra-processed in the NOVA system. Even when notable variations exist between products considered ultra-processed^39,40^, the NOVA system implies that all products classified as such are equally unhealthy. However, herbs and spices can contain bioactive compounds, such as flavonoids and other polyphenols, known for their antioxidative properties^41,42^. This was also evidenced in our analysis as a clear cluster (cluster 2 in Fig. 2e) resulting from phytochemicals derived from spices such as pepper, which were added to the products made from protein concentrates or isolates. This highlights an issue with the NOVA classification system regarding PBPR foods, as it bases the definition of ultra-processed on the processing technique and added ingredients, and the definition of processed on the addition of oil, for example^43^. For instance, tofu and tempeh are categorized as processed according to NOVA. However, if they contain various flavouring ingredients or have been pre-fried, they are labelled as ultra-processed, even though consumers would probably cook and season them similarly at home. In addition, using machine learning to classify food items based on NOVA^44^ highlights this issue: tofu seasoned with herbs receives a high score in FoodProX, indicating that it is ultra-processed, in contrast to unseasoned tofu. To highlight this discrepancy, a tempeh product categorized as ultra-processed actually had the highest abundance of isoflavonoids in our analysis (Fig. 3c). Overall, the various herbs and spices added during the food preparation process may alter the phytochemical composition of the food, and this holds true for both plant-based foods and meat and fish dishes. In our current analysis, all the animal-based products were unseasoned, while some of the PBPR foods were readily seasoned products. If the animal-based products had also been seasoned with herbs, the spices would probably have contributed to a detectable phytochemical content in the poultry, red meat and fish products. However, although spices may add phytochemicals to food products, the primary source of phytochemicals is the plant-based raw material itself.

Ultra-processed foods have been associated with an increased risk for cardiometabolic diseases^45,46^ when considered as a heterogeneous group combining plant-based products with animal-based ones and sugary beverages. However, when subgrouped, plant-based options might not be associated with increased risk for these diseases^9^. Therefore, demeaning PBPR foods by categorizing them as ultra-processed and equating them with foods with less nutritious profiles in terms of bioactive compounds and micronutrients associated with health benefits can be misleading for the consumers. Existing food processing classification systems might categorize products with similar biochemical and nutritional compositions into entirely different classes, or group similar products together. In contrast, the biochemical profiling of soy, as exemplified in our study, provides a more comprehensive approach by distinguishing products based on actual biochemical differences resulting from food processing, which cannot be overlooked when assessing their health impact. For example, fermentation can improve the bioavailability of isoflavonoids, but some of the fermented products in this study would be considered ultra-processed according to NOVA. It is essential to point out that these systems often fail to account for the potential loss of bioactive compounds from the raw material during various processing techniques, and such losses can substantially impact the biochemical composition of the final product, as shown with isoflavonoids in the various soy-based products in this study.

The majority of food products listed in the NOVA ultra-processed category fall into unhealthy convenience food products and sugar-sweetened beverages^47^. However, as highlighted by this study, this category also contains foods, including fermented products, that are rich in phytochemicals and for which there is no scientific support to limit their dietary intake. Ideally, people would consume the presumably healthier ultra-processed foods, such as tempeh, but other factors, such as price and convenience, are important drivers for consumer decisions^48^. Bearing these factors in mind, refining the current classification system could assist in guiding consumers to select nutritionally and phytochemically richer food products. In doing so, the classification should not only consider the intensity of technological processing and the types of added components but also emphasize how much of the original ingredients from the raw material has been removed—that is, the level of refinement—especially in the case of plant-based foods.

We used a state-of-the-art metabolomics approach to determine the comprehensive biochemical composition of various PBPR foods and the effect of processing on bioactive compounds, such as isoflavonoids. Therefore, this study provides valuable information for developing classification systems that would consider not only nutritional content but also overall biochemical composition, including the bioactive phytochemical constituents, when categorizing PBPR foods based on processing. Limitations of this study include the complexity of the biochemical composition of plant-based foods hindering the identification of phytochemicals, which is further influenced by added ingredients, such as spices and herbs. Therefore, thorough analysis of both the raw material and the product would be necessary to expand the phytochemical coverage of our approach.

To conclude, here we showed the ineffectiveness of existing food processing classification systems for PBPR foods, as exemplified with soy-based products and their isoflavonoid content. Our analysis clearly shows how different processing techniques result in very different biochemical compositions that may be relevant from a health-effect perspective; however, these different products may all fall under the ultra-processed food category when using the current classification system. In general, food processing should not be seen as solely harmful as it can also have beneficial effects. Future classification systems should take into account the effect of processing on the biochemical composition of the raw material by considering the loss of bioactive compounds, the formation of new compounds, the value of added ingredients from spices for example, and beneficial processing techniques such as fermentation. Shifting the classification paradigm from the ‘processing level’ to the ‘refinement level’, while also considering certain additives such as sodium, could serve as a more appropriate system for describing PBPR foods in terms of food processing and health aspects. This system should also be better communicated (via food classification) to assist consumers in making healthier food choices and the food industry in developing healthier products.

## Methods

### LC–MS sample collection and preparation

The commonly used processing techniques for soybeans are illustrated in a simplified way in Fig. 4a, along with a simplified workflow of the non-targeted LC–MS method used in this study (Fig. 4b).

PBPR foods (*n* = 168) and unseasoned and unprocessed poultry, red meat and fish (2 minced beef products, 2 chicken strip products, 2 salmon fillets and 2 pork strip products; *n* = 8) were purchased from local markets in Turku, Finland from September to December 2021. Products used in this study are specified in Supplementary Table 2 along with sample codes. The commercially available soy-based products (*n* = 62) included in this study were whole beans, tofu, extruded chunks, tempeh, and meat analogues, such as nuggets, sausages, cold cuts and burger steaks. In this study, extruded chunks include products made with extrusion using soy flour as mentioned in the nutrition label. These products were prepared according to the package instructions, as they would normally be consumed, but without adding any frying oil. Products were then divided into 5 portions and frozen (−20 °C) until further sample preparation. Thawed products were pre-homogenized using Bamix processors (model M133) at full speed (mode 2) or using a pestle and mortar for more complex samples, such as whole beans. Three technical replicates were prepared from 15 plant-based products with complex sample matrices to ensure the efficiency of pre-homogenization, otherwise 1 sample was extracted from each of the remaining 153 PBPR products and 8 poultry, red meat and fish products. Approximately 200 mg of pre-homogenized samples were weighed, and 80% methanol (MeOH) in water was used as the extraction solvent at 400 µl per 100 mg of weighed sample. The samples were vortexed at full speed (5 s, room temperature (RT)) and 3 ceramic beads (2.8 mm; Precellys Ceramic kit) were added before homogenization (30 s^−1^, 1 min × 2; Tissuelyser 2; Qiagen). After homogenization the samples were vortexed (5 s, RT), extracted (15 min, RT) and centrifuged (18,000*g*, 10 min, +4 °C; VWR Mega Star 600R). The collected supernatants (200 µl) were diluted with 80% MeOH (800 µl) to a 1:20 concentration and vortexed (5 s, RT) before filtering with 0.2 µm PTFE filters into HPLC vials with inserts. Quality controls were prepared by pooling 20 µl of each diluted sample and then filtering.

### LC–MS analysis

The LC–MS analysis was performed as a non-targeted metabolite profiling method, as previously described^49^. Ultra-high performance liquid chromatography combined with quadrupole time-of-flight mass spectrometry (LC–QTOF-MS) was used with hydrophilic interaction (HILIC) and reversed-phase (RP) chromatography. Samples were analysed using HILIC (Acquity UPLC BEH Amide, 1.7 µm, 2.1 mm × 100 mm; Waters Corporation) with an Elute UHPLC 1300 coupled with Bruker Impact II QTOF instruments from Bruker Daltonics. The mobile phases consisted of 1:1 acetonitrile in water (solution A) and 9:1 acetonitrile in water (solution B), both containing 20 mM ammonium formate (Sigma-Aldrich). The gradient was 0–2.5 min, 100% B; 2.5–10 min, 100% B to 0% B; 10–10.01 min, 0% B to 100% B; 10.01–12.5 min, 100% B with 0.6 ml min^−1^ flow rate. The injection volume was 2 µl and the sample tray was kept at +4 °C. Electrospray ionization was used with positive and negative mode. The source parameters applied in the analysis were capillary voltage of 3,500 V, end plate offset voltage of 500 V, nebulizer pressure of 45 psi, drying gas flow of 10 l min^−1^ and temperature of 350 °C. In the full scan mode, scan range was set to 50–1600 *m*/*z* and scan rate to 1.67 Hz. Samples were analysed in a randomized order, and quality controls were injected at the beginning of the analysis to prime the system and after every 12 samples to monitor the stability of the LC–MS analysis. Separate data-dependent product ion scans (tandem MS (MS/MS)) using collision energies of 10 eV, 20 eV and 40 eV were acquired in each mode from the quality control and selected samples representing the variety of processed food samples. For the MS/MS scans, scan range was kept at 50–1,600 *m*/*z*, scan rate at 5 Hz, absolute threshold at 48 counts, with a maximum of 4 precursors per cycle with active exclusion enabled for 0.25 min after acquiring 2 spectra. The data were acquired using Bruker Compass HyStar SR 5.0 software (Bruker Daltonics).

For RP chromatography (Zorbax Eclipse XDB-C18, 1.8 µm, 2.1 mm × 100 mm; Agilent Technologies), Agilent 1290 Infinity II UPLC was used, coupled with a 6546 LC–QTOF. Mobile phases consisted of water (solution A) and methanol (solution B), with both containing 0.1% v/v formic acid. The gradient used was 0–10 min, 2–100% B; 10–14.5 min, 100% B; 14.5–14.51 min, 100–2% B; 14.51–16.5 min, 2% B with 0.4 ml min^−1^ flow rate. The injection volume was 2 µl and the sample tray was kept at +4 °C. Electrospray ionization was used with positive and negative mode. Source parameters were set as drying gas flow, 10 l min^−1^; temperature, 325 °C; sheath gas flow, 11 l min^−1^; temperature, 350 °C; nebulizer pressure, 45 psi; capillary voltage, 3,500 V; and nozzle voltage, 1,000 V. In the full scan mode, the scan range was set to 50–1,600 *m*/*z*, scan time to 1.67 Hz and abundance threshold to 150. Samples were analysed in a randomized order, and quality controls were injected at the beginning of the analysis to prime the system and after every 12 samples to monitor the stability of the LC–MS analysis. Collision energies of 10 eV, 20 eV and 40 eV were applied in the separate MS/MS scans for the quality control and selected samples, representing the variety of processed food samples. Parameters for MS/MS scans were an abundance threshold of 200, target of 25,000 counts per spectrum, scan rate of 3.33 Hz, scan range of 50–1,600 *m*/*z*, maximum of 4 precursors per cycle, precursor isolation width of 1.3 Da, active exclusion after 2 spectra and release after 0.25 min. The data acquisition software used was MassHunter Workstation Acquisition 11.0 (Agilent Technologies).

### Data analysis

MS-Dial^50^ (v.4.80) was used for automated peak picking and alignment of the raw data. Peak picking was performed using 0.005 Da for MS tolerance and 0.015 Da for MS/MS tolerance, an *m*/*z* range of 0–2,000 Da, a minimum peak amplitude of 6,000 signal counts, a mass slice width of 0.1 Da, a smoothing level of 3 scans and a minimum peak width of 5 scans. For peak alignment, retention time tolerance was 0.2 min and MS1 tolerance was 0.015 Da. Gap filling by compulsion was also used. The adduct ions for positive mode were [M + H]^+^, [M + NH_4_]^+^, [M + Na]^+^, [M + CH_3_OH + H]^+^, [M + K]^+^, [M + ACN + H]^+^, [M + H − H_2_O]^+^, [M + H − 2H_2_O]^+^, [2M + H]^+^ and [M − NH_4_ + H]^+^; the adduct ions for negative mode were [M − H]^−^, [M − H_2_O − H]^−^, [M + Cl]^−^, [2M − H]^−^ and [M + HCOOH − H]^−^. After alignment, the collected metabolite features from positive and negative modes of RP chromatography (49,708 and 19,390, respectively) and HILIC (24,878 and 6,395, respectively) were exported into Microsoft Excel, resulting in a total of 100,371 molecular features. The raw data from combined modes were preprocessed using the notame (v.0.3.0) package^49^, where the quality control samples were used for data drift correction, flagging low-quality features^51^.

### Metabolite identification

From the resulting 100,371 molecular features from HILIC and RP chromatography (positive and negative ionization for both modes), the most abundant 8,000 features based on the maximum average peak areas for each plant-based raw material, and the remaining 1,286 features containing MS/MS data resulting in a total of 9,286 features, were selected for further analyses. In-house database and publicly available spectral databases were applied in MS-DIAL for metabolite annotation by comparing *m*/*z*, retention time and MS/MS fragmentation patterns. Agilent MassHunter Qualitative Analysis 10.0 was applied for raw data exploration using extracted ion chromatograms and MS/MS fragmentation spectra.

### Statistical analyses

PCAs for plant-based products and unseasoned poultry, red meat and fish using the selected 9,286 features were prepared using ggbiplot^52^ (v.0.6.2) with log transformation. For soy-based products, SIMCA v.16 (Sartorius Stedim Data Analytics) was used for PCA with Pareto scaling, mean centring and log transformation. *k*-means clustering was performed with ComplexHeatmap^53^ (v.2.18.0) using six clusters with *z*-normalized data, and the number of clusters was selected based on visual examination. The Sankey diagram was made using the SankeyMATIC online tool (https://www.sankeymatic.com). The *t*-distributed stochastic neighbour embedding for plant-based products was performed using unit variance scaling and perplexity of five with the pcaMethods (v.1.94.0) and Rtsne (v.0.17) packages. The boxplots for isoflavonoids in soy-based products (Supplementary Fig. 2) were generated using ggplot^52^ (v.3.5.0). R v.4.2.1 (ref. ^54^) was used.

### Reporting summary

Further information on research design is available in the Nature Portfolio Reporting Summary linked to this article.

## Supplementary information

Supplementary Information Supplementary Tables 1 and 2 and Figs. 1–3.

Reporting Summary

Supplementary Data 1 Supplementary dataset.

## Data Availability

The data supporting the findings presented in this study are available within the article and its Supplementary Information.

## Code availability

Code for data preprocessing is available at https://www.github.com/hanhineva-lab/notame.
