# Supplementary material for: Existing food processing classifications overlook the phytochemical composition of processed plant-based protein-rich foods
Source: Nat Food. 2025 Mar 24;6(5):503–12. doi: 10.1038/s43016-025-01148-5 (PMC12116385; doi:10.1038/s43016-025-01148-5)
Supplement: Supplementary file 1 — Supplementary Tables 1 and 2 and Figs. 1–3. [file 43016_2025_1148_MOESM1_ESM.pdf]

nature food

Supplementary information https://doi.org/10.1038/s43016-025-01148-5

Existing food processing classifications overlook the phytochemical composition of processed plant-based protein-rich foods

In the format provided by the authors and unedited

Supplementary information for *"Existing food processing classifications overlook the phytochemical composition of processed plant-based protein-rich foods"* by Raita et al.

Contents

Supplementary Table 1. **Nutritional information for the analyzed plant-based products and poultry, red meat, and fish**.

Supplementary Table 2. **List of the individual plant-based protein-rich products analyzed in the study (n = 168) and their product categories.**

Supplementary Figure 1. **t-Distributed stochastic neighbor embedding (t-SNE) analysis for plant-based products**.

Supplementary Figure 2. **Isoflavonoid abundances in soy-based products.**

Supplementary Figure 3. **Isoflavonoid percentage distributions in soy-based products.**

Supplementary Tables

Supplementary Table 1. ***Nutritional information for the analyzed plant-based products and poultry, red meat, and fish****. Nutritional information of plant-based products (beans, extruded chunks, minced & pulled products, tofu, tempe, cold cuts, nuggets, balls, steaks & other, and sausages) and minced beef, chicken strips, and salmon fillets obtained from packaging labels. Values are presented as mean values of specific product categories with standard deviations for grams or kcals for 100 grams of product. Nutritional information for pork strips is obtained from the national Food Composition Database in Finland for Pork, average, as there were no nutritional information provided in the packages.(1. Accessed on November 18, 2024, http://www.fineli.fi/fineli/en/).*

PLANT-BASED PRODUCT CATEGORIES	ENERGY (KCAL)/ 100 G	TOTAL FAT (G)	SATURATED FAT (G)	CARBOHYDRATE (G)	SUGAR (G)	FIBER (G)	PROTEIN (G)	SALT (G)
**Beans (n=22)**	90.6 ± 24.7	1.3 ± 1.7	0.2 ± 0.2	10.4 ± 3.1	1.0 ± 1.2	5.9 ± 1.6	6.8 ± 2.5	0.4 ± 0.3
**Extruded chunks (n=10)**	339.7 ± 32.4	3.2 ± 2.8	0.5 ± 0.6	26.4 ± 17.0	4.8 ± 2.8	8.8 ± 4.7	49.2 ± 17.7	0.4 ± 0.8
**Minced & Pulled Products (n=29)**	208.9 ± 49.4	9.9 ± 4.6	2.1 ± 2.3	9.1 ± 5.3	2.4 ± 2.6	4.6 ± 2.5	19.3 ± 6.6	1.3 ± 0.5
**Tofu (n=16)**	144.2 ± 62.6	8.2±4.2	1.1±0.7	2.4±1.7	0.4±0.3	0.5±0.7	15.0±6.5	0.2 ± 0.4
**Tempe (n=14)**	196.9 ± 56.1	8.5 ± 7.5	1.3 ± 1.1	12.0 ± 10.5	2.2 ± 2.3	4.8 ± 3.1	17.0 ± 2.1	0.2 ± 0.3
**Cold cuts (n=6)**	192.7 ± 20.1	10.0 ± 3.9	1.8 ± 1.9	6.2 ± 1.4	1.4 ± 0.8	1.4 ± 0.8	22.2 ± 8.7	2.1 ± 0.4
**Nuggets (n=9)**	237.1 ± 29.4	12.3 ± 3.5	1.4 ± 0.9	18.3 ± 3.9	1.4 ± 1.9	5.2 ± 1.4	11.3 ± 2.4	1.2 ± 0.2
**Balls (n=20)**	236.0 ± 45.1	12.3 ± 4.2	1.5 ± 1.3	17.9 ± 7.2	2.1 ± 1.3	6.0 ± 2.0	11.1 ± 3.8	1.2 ± 0.3
**Steaks & Others (n=33)**	215.7 ± 61.5	11.4 ± 5.1	2.4 ± 3.5	12.2 ± 9.2	1.9 ± 2.1	4.8 ± 1.7	15.2 ± 7.7	1.2 ± 0.4
**Sausages (n=9)**	191.6 ± 47.4	12.5 ± 3.9	4.2 ± 2.7	8.4 ± 4.9	1.8 ± 0.6	2.9 ± 1.6	10.1 ± 3.5	1.6 ± 0.3
**POULTRY, RED MEAT, AND FISH**								
**Minced beef (n=2)**	189.5 ± 19.5	12.5 ± 2.5	6.5 ± 1.4				19.5 ± 0.5	0.2 ± 0.01
**Chicken strips (n=2)**	111.5 ± 0.5	4.0 ± 0.0	1.1 ± 0.1				19.0 ± 0.0	0.2 ± 0.03
**Salmon fillets (n=2)**	224.0 ± 0.0	16.0 ± 0.0	3.0 ± 0.0				20.0 ± 0.0	0.1 ± 0.0
**Pork strips[1] (n=2)**	195.0	13.4	4.7				18.9	0.2

Supplementary Table 2. ***List of the individual plant-based protein-rich products analyzed in the study (n = 168) and their product categories****. The product types of individual products are shown with additional information regarding seasoning or specified processing techniques. Sample codes and the raw material protein types (whole bean/plant, extruded chunks, tofu, tempe, and protein concentrates/isolates) are marked in parentheses.*

Beans (n=22)	Extruded chunks (n=10)	Minced & Pulled (n=29)	Soy tofu (n=16)	Tempe (n=14)	Cold cuts (n=6)	Nuggets (n=9)	Balls (n=20)	Steaks & Others (n=33)	Sausages (n=9)
**Edamame**, frozen (R12, Whole bean/plant)	**Soy mince**, extruded (D1, Extruded chunks)	**Soy mince**, seasoned (M15, Extruded chunks)	**Tofu**, soft (T1, Tofu)	**Soy tempe** (TE10, Tempe)	**Soy-based bacon** (C1, Protein concentrates/isolates)	**Soy nuggets**, seasoned (N1, Protein concentrates/isolates)	**Soy balls** (B10, Protein concentrates/isolates)	**Soy burger steak** (010, Protein concentrates/isolates)	**Soy sausage**, seasoned (S1, Protein concentrates/isolates)
**Edamame**, frozen (R17, Whole bean/plant)	**Soy mince**, extruded (D5, Extruded chunks)	**Soy chunks**, seasoned (M22, Protein concentrates/isolates)	**Tofu**, fried (T10, Tofu)	**Soy tempe**, smoked (TE11, Tempe)	**Soy cold cuts**, seasoned (C2, Protein concentrates/isolates)	**Soy nuggets** (N4, Protein concentrates/isolates)	**Soy balls**, seasoned (B11, Protein concentrates/isolates)	**Soy-based "chicken" wings** (O11, Protein concentrates/isolates)	**Soy sausage** (S7, Protein concentrates/isolates)
**Chickpeas**, steamed (R1, Whole bean/plant)	**Soy chunks**, extruded (D6, Extruded chunks)	**Soy chunks**, seasoned (M23, Protein concentrates/isolates)	**Tofu**, smoked (T11, Tofu)	**Soy tempe**, fried (TE12, Tempe)	**Wheat cold cuts** (C3, Protein concentrates/isolates)	**Soy nuggets** (N5, Protein concentrates/isolates)	**Soy balls** (B20, Protein concentrates/isolates)	**Soy steak** (O12, Protein concentrates/isolates)	**Pea sausage** (S2, Protein concentrates/isolates)
**Peas**, salt-brine (R11, Whole bean/plant)	**Soy strips**, extruded (D7, Extruded chunks)	**Soy chunks** (M28, Protein concentrates/isolates)	**Tofu**, soft (T13, Tofu)	**Soy tempe** (TE13, Tempe)	**Wheat cold cuts** (C4, Protein concentrates/isolates)	**Soy nuggets** (N6, Protein concentrates/isolates)	**Pea balls** (B1, Protein concentrates/isolates)	**Soy chunks**, seasoned (O13, Protein concentrates/isolates)	**Sun flower seed sausage** (S3, Whole bean/plant)
**Black peas**, water (R10, Whole bean/plant)	**Fava beans**, extruded (D10**,** Whole bean/plant)	**Soy mince**, seasoned (M27, Protein concentrates/isolates)	**Tofu** (T12, Tofu)	**Soy tempe** (TE2, Tempe)	**Pea cold cuts** (C5, Protein concentrates/isolates)	**Soy nuggets** (N9, Protein concentrates/isolates)	**Pea balls**, seasoned (B12, Protein concentrates/isolates)	**Soy chunks**, seasoned (O14, Protein concentrates/isolates)	**Pea sausage** (S4, Protein concentrates/isolates)
**Fava beans**, smoked, salt-brine (R13, Whole bean/plant)	**Fava beans**, extruded (D2, Extruded chunks)	**Soy mince**, seasoned (M29, Protein concentrates/isolates)	**Tofu**, fried (T15, Tofu)	**Soy tempe**, seasoned (TE3, Tempe)	**Wheat cold cuts** (C6, Protein concentrates/isolates)	**Wheat nuggets** (N2, Protein concentrates/isolates)	**Pea balls** (B13, Protein concentrates/isolates)	**Soy chunks**, seasoned (O15, Protein concentrates/isolates)	**Fava bean sausage** (S5, Protein concentrates/isolates)
**White beans**, salt-brine (R14, Whole bean/plant)	**Peas**, crushed (D3**,** Whole bean/plant)	**Fava beans strips**, fermented (M6**,** Whole bean/plant)	**Tofu**, seasoned (T16, Tofu)	**Soy tempe**, seasoned (TE4, Tempe)		**Pea nuggets** (N3, Protein concentrates/isolates)	**Chickpea falafel** (B14, Whole bean/plant)	**Soy chunks**, seasoned (O16, Protein concentrates/isolates)	**Pea sausage** (S6, Protein concentrates/isolates)
**Kidney beans**, salt-brine (R15, Whole bean/plant)	**Peas**, extruded (D4, Extruded chunks)	**Fava beans strips**, fermented (M7**,** Whole bean/plant)	**Tofu** (T14, Tofu)	**Soy tempe**, seasoned (TE6, Tempe)		**Pea nuggets** (N7, Protein concentrates/isolates)	**Chickpea falafel** (B15, Whole bean/plant)	**Soy-based "fish" sticks** (O17, Protein concentrates/isolates)	**Pea sausage** (S8, Protein concentrates/isolates)
**Red lentils**, salt-brine (R16, Whole bean/plant)	**Peas**, extruded (D8, Extruded chunks)	**Peas strips**, seasoned (M1, Protein concentrates/isolates)	**Tofu**, seasoned (T3, Tofu)	**Soy tempe** (TE7, Tempe)		**Rice nuggets** (N8, Protein concentrates/isolates)	**Chickpea falafel** (B16, Whole bean/plant)	**Soy burger steak** (O23, Protein concentrates/isolates)	**Sun flower seed sausage** (S9, Protein concentrates/isolates)
**Black beans**, salt-brine (R18, Whole bean/plant)	**Fava beans**, crushed (D9**,** Extruded chunks)	**Fava beans**, minced (M5**,** Whole bean/plant)	**Tofu** (T2, Tofu)	**Fava bean tempe**, seasoned (TE1, Tempe)			**Chickpea falafel** (B17, Whole bean/plant)	**Soy burger steak** (O25**,** Whole bean/plant)	
**Chickpeas**, salt-brine (R19, Whole bean/plant)		**Peas,** minced (M4, Protein concentrates/isolates)	**Tofu** (T4, Tofu)	**Fava bean tempe** (TE14, Tempe)			**Pea balls** (B18, Protein concentrates/isolates)	**Soy seitan** (O31, Protein concentrates/isolates)	
**Peas**, salt-brine (R2, Whole bean/plant)		**Pea strips**, seasoned (M3, Protein concentrates/isolates)	**Tofu** (T5, Tofu)	**Fava bean tempe**, seasoned (TE5, Tempe)			**Pea balls** (B19, Protein concentrates/isolates)	**Soy burger steak** (O26, Protein concentrates/isolates)	
**Butter beans**, water (R20, Whole bean/plant)		**Pea strips**, seasoned (M2, Protein concentrates/isolates)	**Tofu** (T6, Tofu)	**Pea tempe** (TE8, Tempe)			**Chickpea falafel** (B2, Whole bean/plant)	**Soy burger steak** (O33, Protein concentrates/isolates)	
**Green lentils**, water (R21, Whole bean/plant)		**Oats**, pulled (M8**,** Whole bean/plant)	**Tofu**, seasoned (T8, Tofu)	**Fava bean tempe** (TE9, Tempe)			**Oat balls** (B3, Whole bean/plant)	**Pea steak** (O1, Protein concentrates/isolates)	
**Kidney beans**, water (R22, Whole bean/plant)		**Oats**, pulled, seasoned (M13**,** Whole bean/plant)	**Tofu**, seasoned (T9, Tofu)				**Oat balls**, seasoned (B4, Whole bean/plant)	**Pea burger steak** (O18, Protein concentrates/isolates)	
**Fava beans**, steamed (R3, Whole bean/plant)		**Oats**, pulled, seasoned (M12, Whole bean/plant)	**Tofu** (T7, Tofu)				**Fava bean balls** (B5, Whole bean/plant)	**Wheat "chicken" fingers** (O19, Protein concentrates/isolates)	
**Borlotti beans**, water (R4, Whole bean/plant)		**Oats**, pulled (M10**,** Whole bean/plant)					**Fava bean balls** (B6, Whole bean/plant)	**Wheat "fish" steak** (O2, Protein concentrates/isolates)	

**Butter beans**, water (R5, Whole bean/plant)	**Oats**, pulled, seasoned (M11**,** Whole bean/plant)	**Chickpea falafel** (B7, Whole bean/plant)	**Wheat seitan** (O20, Protein concentrates/isolates)
**Borlotti beans**, water (R6, Whole bean/plant)	**Peas**, minced (M9, Protein concentrates/isolates)	**Fava bean falafel** (B8, Whole bean/plant)	**Wheat seitan** (O28, Protein concentrates/isolates)
**White beans**, water (R7, Whole bean/plant)	**Peas**, minced (M14, Protein concentrates/isolates)	**Pea balls** (B9, Protein concentrates/isolates)	**Pea burger steak** (O21, Protein concentrates/isolates)
**Green lentils**, water (R8, Whole bean/plant)	**Wheat strips**, seasoned (M18, Protein concentrates/isolates)		**Pea burger steak** (O22, Protein concentrates/isolates)
**Red lentils**, water (R9, Whole bean/plant)	**Wheat**, minced (M17, Protein concentrates/isolates)		**Chickpea steak** (O24**,** Whole bean/plant)
	**Wheat strips** (M19, Protein concentrates/isolates)		**Chickpea burger steak** (O27, Whole bean/plant)
	**Peas**, minced (M16, Protein concentrates/isolates)		**Wheat seitan** (O32, Protein concentrates/isolates)
	**Peas**, minced (M21, Protein concentrates/isolates)		**Wheat seitan** (O29, Protein concentrates/isolates)
	**Peas**, minced (M20, Protein concentrates/isolates)		**Pea burger steak** (O3, Protein concentrates/isolates)
	**Fava beans,** fermented, seasoned (M25, Whole bean/plant)		**Wheat "chicken" wings** (O30, Protein concentrates/isolates)
	**Fava beans**, fermented, seasoned (M26**,** Whole bean/plant)		**Lentil steak** (O9**,** Whole bean/plant)
	**Peas**, minced (M24, Protein concentrates/isolates)		**Oat rings,** seasoned (O4, Whole bean/plant)
			**Wheat steak** (O6, Protein concentrates/isolates)
			**Oat rings**, seasoned (O5, Whole bean/plant)
			**Pea steak** (O7, Whole bean/plant)
			**Black bean steak** (O8**,** Whole bean/plant)

Supplementary Figures

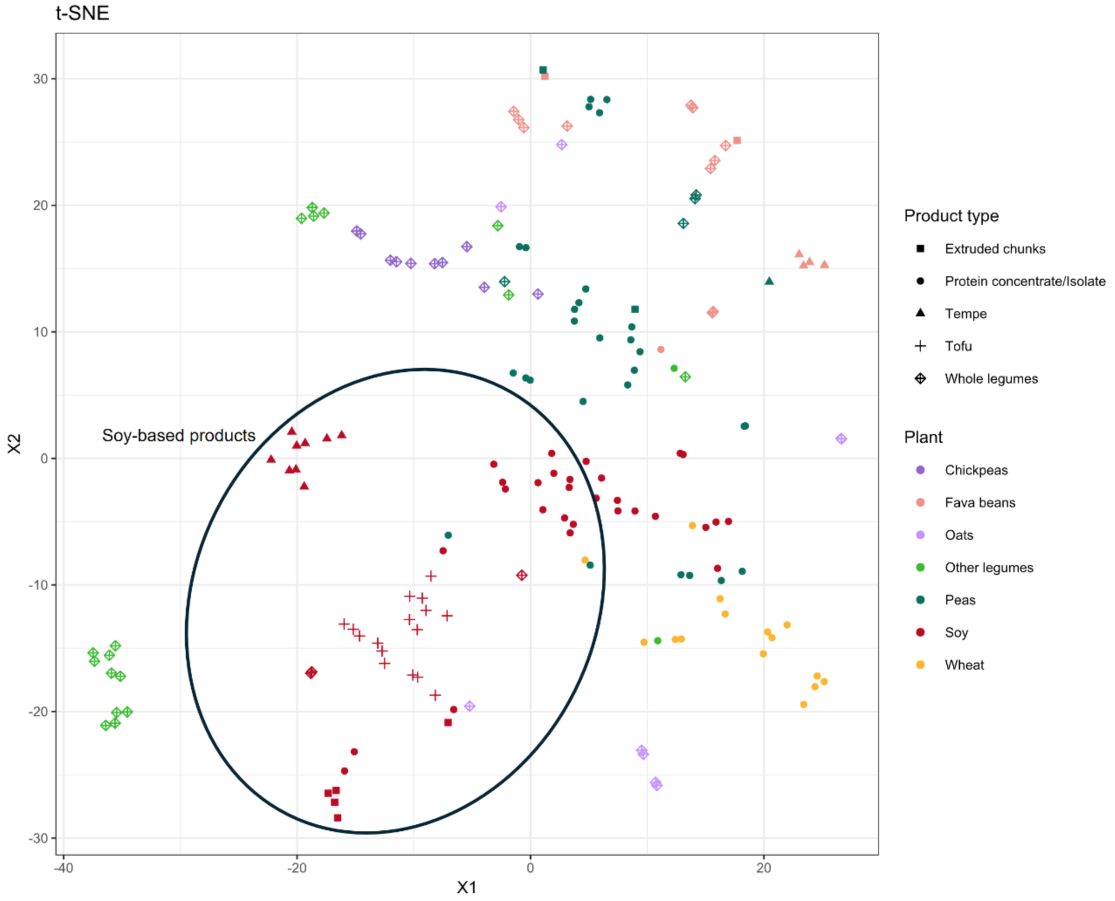

*Supplementary Figure 1. **t-Distributed stochastic neighbor embedding (t-SNE) analysis for plant-based products**. t-SNE analysis made with plant-based products (categorized as protein types according to the form of the raw material used: extruded chunks (n = 9), whole bean/plant (n = 54), tofu (n = 16), tempe (n = 14), and products made with protein concentrates/isolates (n = 75); plant: (chickpeas (n = 10), fava beans (n = 18), oats (n = 9), other legumes (n = 19), peas (n = 35), soy (n = 62), wheat (n = 15)) using molecular features obtained from LC-MS data (n = 9286). Soy-based tofu, tempe, whole beans, and extruded chunks separated from other plant-based products.*

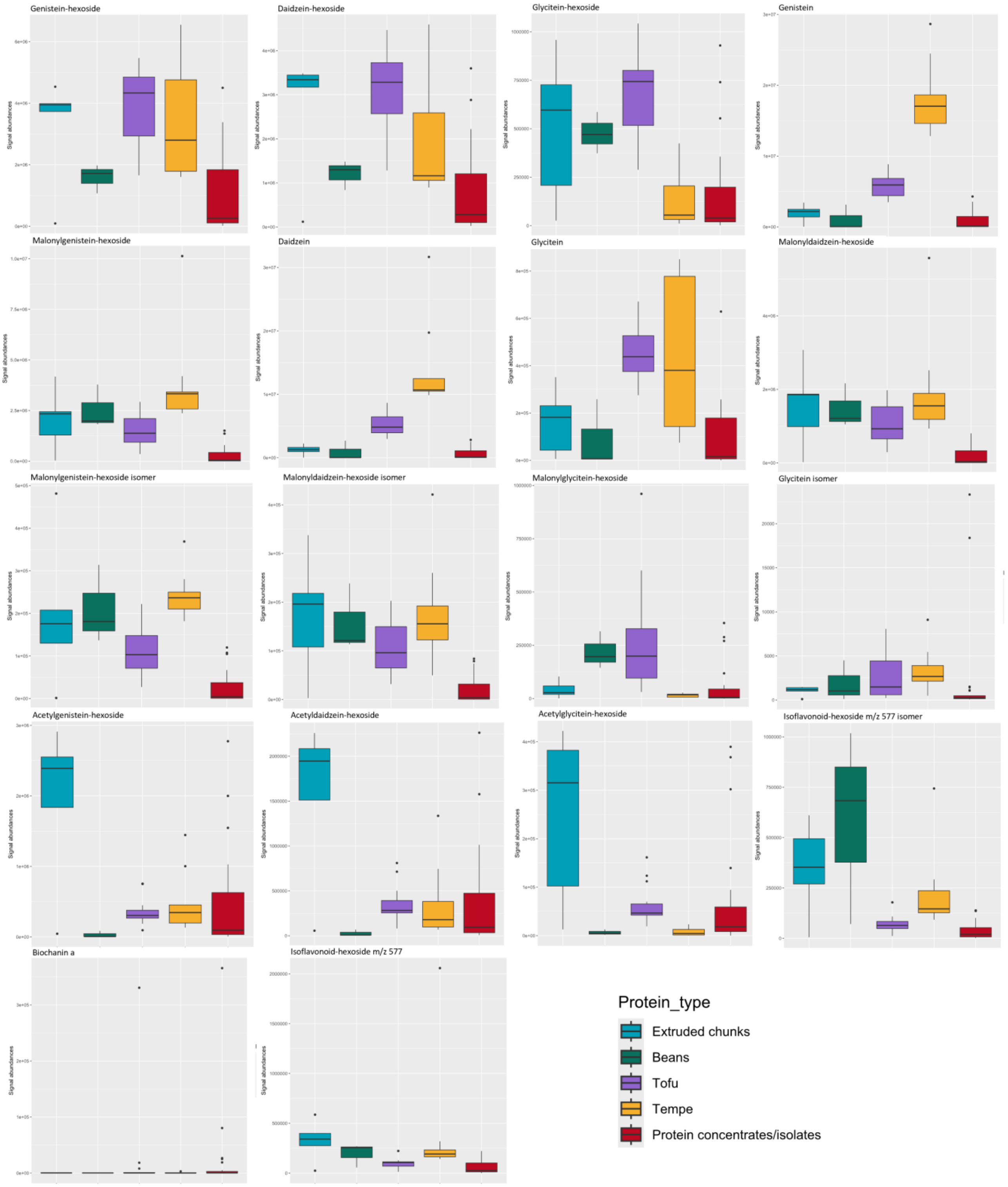

Supplementary Figure 2. ***Isoflavonoid abundances in soy-based products.*** *Isoflavonoid compounds in different soy-based products (categorized as protein types according to the form of the raw material used: extruded chunks (n = 5), beans (n = 3), tofu (n = 16), tempe (n = 9), and products made with protein concentrates/isolates (n = 29)) expressed as signal abundances. The box shows the 25th and 75th percentiles, and the horizontal line represents the median. Whiskers represent the minimum and maximum values, while dots represent outliers.*

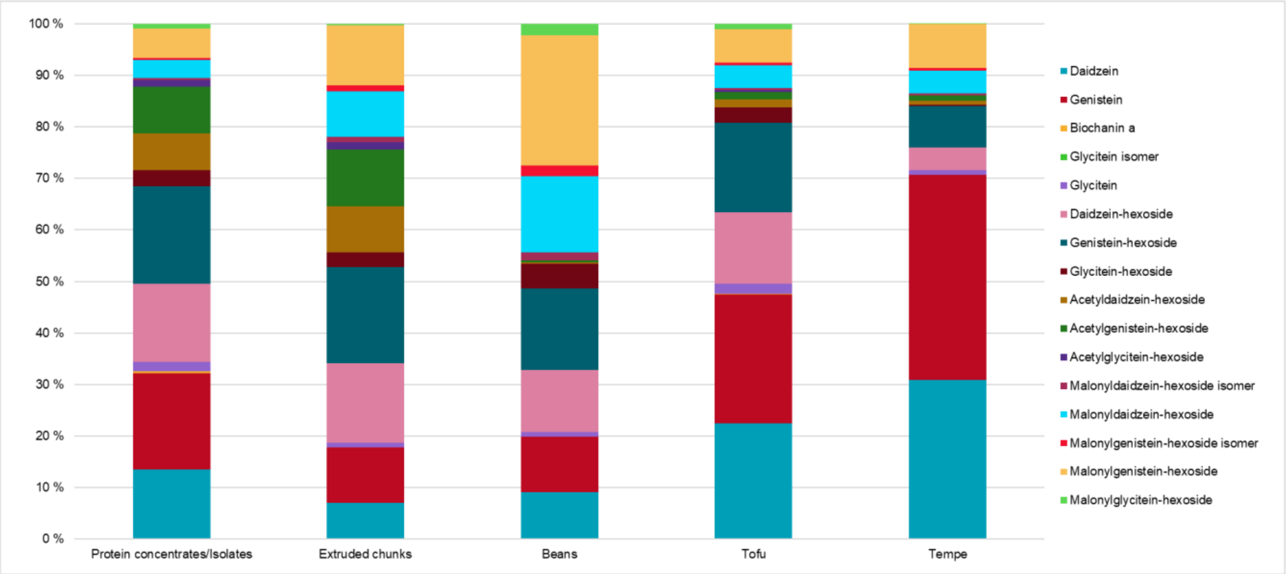

Supplementary Figure 3. ***Isoflavonoid percentage distributions in soy-based products.*** *Isoflavonoid compounds in soy-based products (categorized as protein types according to the form of the raw material used:extruded chunks (n = 5), beans (n = 3), tofu (n = 16), tempe (n = 9), and products made with protein concentrates/isolates (n = 29)) expressed as percentages based on the signal abundances.*
