# Supplementary material for: Existing food processing classifications overlook the phytochemical composition of processed plant-based protein-rich foods
Source: Nat Food. 2025 Mar 24;6(5):503–12. doi: 10.1038/s43016-025-01148-5 (PMC12116385; doi:10.1038/s43016-025-01148-5)
Supplement: Supplementary file 2 — Reporting Summary [file 43016_2025_1148_MOESM2_ESM.pdf]

nature portfolio

Corresponding author(s): Kati Hanhineva

Last updated by author(s): Feb 12, 2025

Reporting Summary

Nature Portfolio wishes to improve the reproducibility of the work that we publish. This form provides structure for consistency and transparency in reporting. For further information on Nature Portfolio policies, see our Editorial Policies and the Editorial Policy Checklist.

Statistics

For all statistical analyses, confirm that the following items are present in the figure legend, table legend, main text, or Methods section.

n/a	Confirmed	
☐	☒	The exact sample size (*n*) for each experimental group/condition, given as a discrete number and unit of measurement
☐	☒	A statement on whether measurements were taken from distinct samples or whether the same sample was measured repeatedly
☒	☐	The statistical test(s) used AND whether they are one- or two-sided<br>*Only common tests should be described solely by name; describe more complex techniques in the Methods section.*
☒	☐	A description of all covariates tested
☒	☐	A description of any assumptions or corrections, such as tests of normality and adjustment for multiple comparisons
☐	☒	A full description of the statistical parameters including central tendency (e.g. means) or other basic estimates (e.g. regression coefficient) AND variation (e.g. standard deviation) or associated estimates of uncertainty (e.g. confidence intervals)
☒	☐	For null hypothesis testing, the test statistic (e.g. *F*, *t*, *r*) with confidence intervals, effect sizes, degrees of freedom and *P* value noted<br>*Give P values as exact values whenever suitable.*
☒	☐	For Bayesian analysis, information on the choice of priors and Markov chain Monte Carlo settings
☐	☒	For hierarchical and complex designs, identification of the appropriate level for tests and full reporting of outcomes
☒	☐	Estimates of effect sizes (e.g. Cohen's *d*, Pearson's *r*), indicating how they were calculated

Our web collection on statistics for biologists contains articles on many of the points above.

Software and code

Policy information about availability of computer code

Data collection	The data was collected with Bruker Compass HyStar SR 5.0 and MassHunter Workstation Acquisition 11.0 softwares.
Data analysis	The data analysis was performed with MS-DIAL version 4.80, R Version 4.2.1, SIMCA version 16, Microsoft Excel version 2501, and SankeyMATIC online tool (https://www.sankeymatic.com, accessed August, 2024). R-packages used in data and statistical analyses include notame (Version 0.3.0), ggbiplot (Version 0.6.2), ComplexHeatmap (Version 2.18.0), pcaMethods (Version 1.94.0), Rtsne (Version 0.17), and ggplot (Version 3.5.0). Code for data preprocessing available at https://www.github.com/hanhineva-lab/notame.

For manuscripts utilizing custom algorithms or software that are central to the research but not yet described in published literature, software must be made available to editors and reviewers. We strongly encourage code deposition in a community repository (e.g. GitHub). See the Nature Portfolio guidelines for submitting code & software for further information.

Data

Policy information about availability of data

All manuscripts must include a data availability statement. This statement should provide the following information, where applicable:

- Accession codes, unique identifiers, or web links for publicly available datasets
- A description of any restrictions on data availability
- For clinical datasets or third party data, please ensure that the statement adheres to our policy

The data supporting the findings presented in this study are available within the paper and its supplementary information files.

Human research participants

Policy information about studies involving human research participants and Sex and Gender in Research.

Reporting on sex and gender	not applicable
Population characteristics	not applicable
Recruitment	not applicable
Ethics oversight	not applicable

Note that full information on the approval of the study protocol must also be provided in the manuscript.

Field-specific reporting

Please select the one below that is the best fit for your research. If you are not sure, read the appropriate sections before making your selection.

☒ Life sciences ☐ Behavioural & social sciences ☐ Ecological, evolutionary & environmental sciences

For a reference copy of the document with all sections, see nature.com/documents/nr-reporting-summary-flat.pdf

Life sciences study design

All studies must disclose on these points even when the disclosure is negative.

Sample size	Three replicates of 15 samples with more complex matrices were prepared to ensure the efficiency of pre-homogenization during sample preparation. The extraction process including homogenization was found to be sufficient for analyzing the biochemical composition of individual PBPR food products (n=186) with varying product types and meat (n=8) products. No sample size calculations were performed. Sample size as food products (n = 186) was chosen by selecting all relevant food products available at the market at the time of purchase (September to December 2021).
Data exclusions	No data was excluded from this study.
Replication	The non-targeted metabolomics method used has been previously published (Klåvus et al. 2020) and can be used to replicate this study. We used commercially available products.
Randomization	Sample order was randomized for the LC-MS analysis.
Blinding	Blinding was not relevant in this study as the samples were food products and no human participants were involved.

Reporting for specific materials, systems and methods

We require information from authors about some types of materials, experimental systems and methods used in many studies. Here, indicate whether each material, system or method listed is relevant to your study. If you are not sure if a list item applies to your research, read the appropriate section before selecting a response.

Materials & experimental systems

n/a	Involved in the study
☒	☐ Antibodies
☒	☐ Eukaryotic cell lines
☒	☐ Palaeontology and archaeology
☒	☐ Animals and other organisms
☒	☐ Clinical data
☒	☐ Dual use research of concern

Methods

n/a	Involved in the study
☒	☐ ChIP-seq
☒	☐ Flow cytometry
☒	☐ MRI-based neuroimaging
